# Elevated Preoperative Serum Gamma-glutamyltranspeptidase Predicts Poor Prognosis for Hepatocellular Carcinoma after Liver Transplantation

**DOI:** 10.1038/srep28835

**Published:** 2016-07-06

**Authors:** Shun-Jun Fu, Qiang Zhao, Fei Ji, Mao-Gen Chen, Lin-Wei Wu, Qing-Qi Ren, Zhi-Yong Guo, Xiao-Shun He

**Affiliations:** 1 Organ Transplant Center, the First Affiliated Hospital, Sun Yat-sen University, Guangzhou 510080, P. R. China; 2 Guangdong Provincial Key Laboratory of Organ Donation and Transplant Immunology, the First Affiliated Hospital, Sun Yat-sen University, Guangzhou 510080, P. R. China; 3 Guangdong Provincial International Cooperation Base of Science and Technology (Organ Transplantation), the First Affiliated Hospital, Sun Yat-sen University, Guangzhou 510080, P. R. China

## Abstract

Gamma-glutamyltransferase (γ-GGT) is a membrane-bound enzyme that is involved in biotransformation, nucleic acid metabolism, and tumourigenesis. Elevated serum γ-GGT levels are related to an increased cancer risk and worse prognosis in many cancers. In the present study, we evaluated the prognostic value of preoperative serum γ-GGT in patients with hepatocellular carcinoma (HCC) who underwent liver transplantation (LT). A total of 130 HCC patients after LT were included in the study. The optimal cut-off value of γ-GGT was 128U/L by receiver operating characteristic analysis, with a sensitivity and specificity of 60.0% and 72.9%, respectively. Elevated preoperative serum γ-GGT was significantly associated with high alpha-fetoprotein (AFP), large tumor size, and macro- and micro-vascular invasion. The 1-, 3-, 5-year disease-free survival (DFS) and overall survival (OS) rates of HCC patients in the γ-GGT > 128U/L group were poorer than those in the γ-GGT ≤ 128U/L group. Stratification analysis revealed that γ-GGT exhibited a greater predictive value for DFS and OS in HCC patients beyond the Milan criteria and no macro-vascular invasion. In conclusion, elevated preoperative serum γ-GGT was significantly associated with advanced tumor stage and aggressive tumor behaviors, and serum γ-GGT can be considered as a prognostic factor for HCC patients after LT, especially for patients beyond the Milan criteria or without macro-vascular invasion.

Hepatocellular carcinoma (HCC) is the fifth-most common cancer and the second leading cause of cancer-related death worldwide, with an estimated global incidence of 782,000 new cases and nearly 746,000 deaths in 2012^1^. Liver transplantation (LT) has been considered as an optimal radical therapy for selected patients with HCC, especially since the introduction of the Milan criteria in 1996^2^. The Milan criteria are recognized as the standard selection criteria of transplant candidates for HCC patients. The most significant factor affecting long-term outcomes is the high postoperative recurrence rate. Despite the excellent outcomes for HCC patients fulfilling the Milan criteria, nearly 30% of patients experience tumor recurrence^3^. Some clinico- pathological factors, including tumor size, macro-vascular invasion, and poor differentiation as well as some molecular biomarkers, such as alpha-fetoprotein (AFP) and glypican-3 (GPC3), have been identified as prognostic predictors of HCC^3^,^4^,^5^,^6^. However, the identification of additional prognostic factors to identify patients with HCC who are at high risk of recurrence and HCC-related death is critical to improve the prognosis of these patients through novel treatments and clinical decision-making surveillance schedules.

Gamma-glutamyltransferase (γ-GGT) is a membrane-bound enzyme involved in the glutathione (GSH) metabolism^7^. Serum γ-GGT is a well-known marker of hepatic injury; elevated levels can result from alcohol consumption, acute and chronic liver disease and oxidative stress^8^,^9^,^10^. Similarly, elevated γ-GGT expression had been observed in various tumors^11^,^12^. Moreover, a study has suggested that γ-GGT plays an important role in carcinogenesis by exerting pro-oxidant effects at the membrane surface level and in the extracellular microenvironment and by promoting the release of free iron from transferrin^13^. GGT is involved in the prevention of apoptosis and the maintenance of proliferation through the production of hydrogen peroxide in U937 lymphoma cells^14^. GGT is essential to overcoming the toxicity of the drug by providing additional cysteine and can promote the drug resistance of cancer cells^15^. Therefore, GGT plays an important role with respect to the risk of cancer development, tumor progression, invasion and anticancer drug resistance^15^,^16^,^17^. Recently, serum γ-GGT has attracted attention as a prognostic marker for several human malignancies, including renal cell carcinoma, ovarian cancer and esophageal squamous cell carcinoma^18^,^19^,^20^. With respect to HCC, elevated serum γ-GGT has been reported to be a biomarker of poor prognosis after hepatectomy, transcatheter arterial chemoembolization (TACE) and radiofrequency ablation (RFA)^21^,^22^,^23^,^24^. However, the prognostic role of γ-GGT in HCC patients undergoing liver transplantation has not been reported thus far. We hypothesized that γ-GGT plays an important role in HCC progression and metastasis, and that it may represent a prognostic factor for HCC patients after LT. To test this hypothesis, the association of preoperative serum γ-GGT with survival was investigated in 130 HCC patients who underwent LT.

## Materials and Methods

### Patients

We included 130 patients with HCC who underwent liver transplantation at the Organ Transplant Center of The First Affiliated Hospital of Sun Yat-sen University between January 2008 and May 2013. The diagnosis of HCC was made based on postoperative pathological examination. Prior to LT, a complete physical examination, blood test, electrocardiograph (ECG), abdominal ultrasound, computed tomography (CT) or magnetic resonance imaging (MRI), and chest X-ray or CT scan were performed. All patients have complete clinical, pathological, laboratory and follow-up data. All patients gave written informed consent to be in the study, and approval for the study was obtained from the Ethics Committee of the First Affiliated Hospital of Sun Yat-sen University. The methods were carried out in accordance with the approved guidelines.

### Measurement of serum γ-GGT levels

As part of routine clinical testing, blood samples for the evaluation of serum γ-GGT levels were collected from peripheral venous blood on the day before surgery. Serum γ-GGT concentrations were analyzed with a commercially available enzyme-coupled assay kit (Beijing Homa Biological Co., Ltd., Beijing, China) as previously described^21^. The normal value range of γ-GGT was between 2 and 50 U/L in our hospital.

### Clinical management and follow-up

All patients were received in a classic orthotropic or modified piggyback fashion using well-described standard techniques^25^. An immunosuppressive regimen with anti-IL-2 receptor antibody (basiliximab) induction and tacrolimus and mycophenolate mofetil (MMF) maintenance was administered in these patients. The initial dose of tacrolimus was 0.04 mg/kg/d and the target trough tacrolimus level was 8–10 ng/ml within the first 3 months and 6–8 ng/ml thereafter. MMF was administered at 500–750 mg twice a day. A prophylactic regimen of lamivudine/entecavir and low-dose hepatitis B immunoglobulin was used to prevent hepatitis B virus (HBV) recurrence for patients with pre-transplant HBV infection. Routine tests of the concentrations of tacrolimus and MMF were performed; and immunosuppressive drugs were adjusted based on the drug concentration, liver function, serum biochemistry and blood routine during the procedure at each follow-up. Serum AFP and liver ultrasound were performed at each follow-up. Abdominal CT scan and chest X-ray was performed every 3–6 months or when recurrence was suspected. Recurrence was defined as emergence of clinical, radiological, and/or pathological diagnosis (tissues obtained by ultrasound-guided fine-needle aspiration). Once the recurrence was confirmed, patients were further treated by RFA, TACE, liver resection and/or sorafenib according to the size, number, and location of recurrent tumors as well as liver function.

### Statistical analysis

The statistical analysis was conducted with Statistical Package for Social Science (SPSS) version 19.0 software (Chicago, IL, USA). All continuous variables are expressed as the mean ± standard deviation (SD). The difference between two groups were analyzed using the unpaired Student’s *t* or Mann-Whitney U test for continuous variables, and categorical variables were compared through the chi-square or Fisher’s exact test. A receiver operating characteristic curve (ROC) analysis was used to select the optimal cut-off value of preoperative serum γ-GGT. The survival analysis was performed by the Kaplan-Meier method and compared through the log-rank test. The Cox proportional hazard model was used to determine independent prognostic factors. The disease-free survival (DFS) time was calculated from the date of operation to the date of recurrence or the last follow-up date. The overall survival (OS) time was calculated from the date of operation to the date of death or the last follow-up date. The last follow-up date was October 31^st^, 2015. All statistical tests were 2-sided, and *P* < 0.05 was considered to be statistically significant.

## Results

### Clinical and pathological characteristics

A total of 130 patients were enrolled in this study, including 121 (93.1%, 121/130) male and 9 (6.9%, 9/130) female patients. The median age was 49.5 (ranging from 13 to 72) years. One-hundred and nineteen out of the 130 patients were infected by hepatitis B virus (HBV). On the basis of the Child-Pugh liver function class system, 84 patients were classified as Class A, 36 as Class B and 10 as Class C. Fifty-nine patients (45.4%, 59/130) received various therapies including hepatectomy, RFA, and TACE before transplantation. According to Edmondson-Steiner tumor differentiation stage, there were 86 (66.2%, 86/130) stage I–II and 44 (33.8%, 44/130) stage III–IV patients. Three category standards were used in this study: Milan criteria^2^, the University of California at San Francisco (UCSF) criteria^26^, and Hangzhou criteria^27^. For these patients, 46 (35.4%, 46/130) patients fulfilled the Milan criteria, whereas, 69 (53.1%, 69/130) and 81 (62.3%, 81/130) patients fulfilled the UCSF criteria and Hangzhou criteria, respectively.

### Determination of the cut-off value for elevated γ-GGT by ROC curve

**Figure 1:**
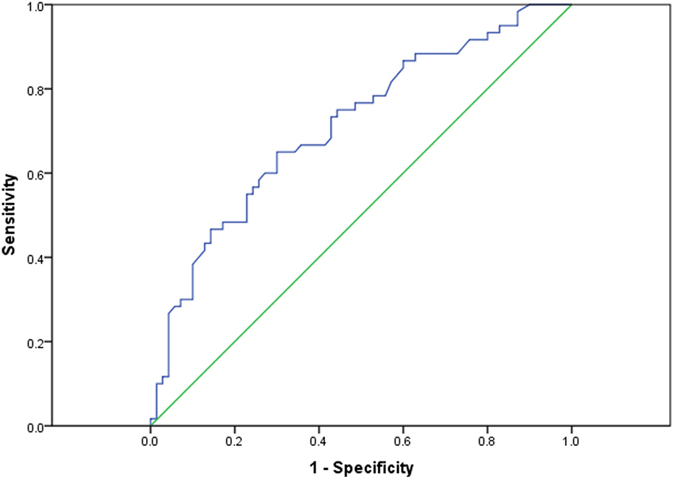
Receiver operating characteristic curves for the determination of the cut-off value for preoperative serum γ-GGT in patients with HCC after liver transplantation.

The ROC curve analysis indicated that the optimal cut-off value of serum γ-GGT was 128 U/L for predicting recurrence. The area under the ROC curve was 0.712, with a 95% confidential interval (CI) of 0.623–0.801 (Fig. 1). It presented a sensitivity of 60.0% and a specificity of 72.9% and was related to the highest Youden Index (sensitivity+specificity-1). Subsequently, all patients were classified into either the γ-GGT ≤ 128 U/L (n = 77) group or the γ-GGT > 128U/L group (n = 53).

### Association of preoperative serum γ-GGT with clinicopathological features

The association between preoperative serum γ-GGT and clinicopathological features is summarized Table 1. Preoperative serum γ-GGT increased with AFP > 400 ng/ml (*P* = 0.002), larger tumor (*P* < 0.001), and macro- (*P* < 0.001) and micro-vascular invasion (*P* = 0.001). A higher proportion of patients with γ-GGT > 128U/L were beyond the Milan criteria (52.4%, 44/84), UCSF criteria (54.1%, 33/61) and Hangzhou criteria (63.3%, 31/49) than those with γ-GGT ≤ 128 U/L (all *P* < 0.001). However, there was no association of γ-GGT levels with gender (*P* = 0.241), age (*P* = 0.538), hepatitis B surface antigen (HBsAg) (*P* = 0.756), Child-Pugh stage (*P* = 0.097), preoperative tumor therapy (*P* = 0.985), tumor number (*P* = 0.249) or Edmondson grading (*P* = 0.248).

### DFS and OS according to γ-GGT levels

**Figure 2:**
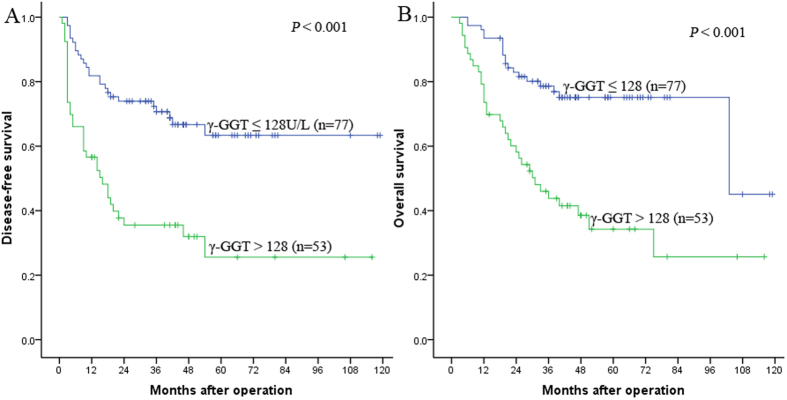
Kaplan-Meier survival curves demonstrating that patients with γ-GGT ≤ 128 U/L exhibited shorter DFS (**A**) and OS (**B**) rates than those with γ-GGT > 128 U/L (all *P* < 0.001, log-rank).

The influence of preoperative serum γ-GGT levels on prognosis was analyzed. The results showed that the 1-, 3-, and 5-year DFS rates were 81.8%, 70.7%, and 63.4% in the γ-GGT ≤ 128 U/L group, and 56.6%, 35.5%, and 25.6% in the γ-GGT > 128 U/L group, respectively (*P* < 0.001, Fig. 2A). Correspondingly, the 1-, 3-, and 5-year OS rates were 93.5%, 78.6%, and 75.1% in the γ-GGT ≤ 128 U/L group, and 73.6%, 43.8%, and 34.3% in the γ-GGT > 128 U/L group, respectively (*P* < 0.001, Fig. 2B). The preoperative serum γ-GGT > 128 U/L was a risk factor for HCC patients who underwent liver transplantation.

### Risk factors for HCC patients’ prognosis

After a median follow-up period of 40.3 (range 3–119) months, 60 (60/130) patients experienced recurrence and 53 (53/130) patients died. For all patients included in this study, the 1-, 3-, and 5-year DFS rates were 71.5%, 56.7%, and 48.7%, respectively. For the same period, the 1-, 3-, and 5-year OS rates were 85.4%, 64.3%, and 58.0%, respectively. A univariate analysis identified the Child-Pugh stage, tumor number, size of largest tumor, macro-vascular invasion, micro-vascular invasion, AFP and γ-GGT as significant risk factors associated with DFS. Furthermore, tumor number, size of largest tumor, macro-vascular invasion, micro-vascular invasion, AFP and γ-GGT were significant risk factors associated with OS (Table 2). Multivariate Cox proportional hazards regression model revealed that Child-Pugh stage, tumor number, size of largest tumor, AFP and γ-GGT were independent risk factors of DFS, and tumor number, size of largest tumor, and γ-GGT were independent risk factors of OS (Table 3).

### Prognostic value of preoperative serum γ-GGT in HCC patients without macro-vascular invasion

**Figure 3:**
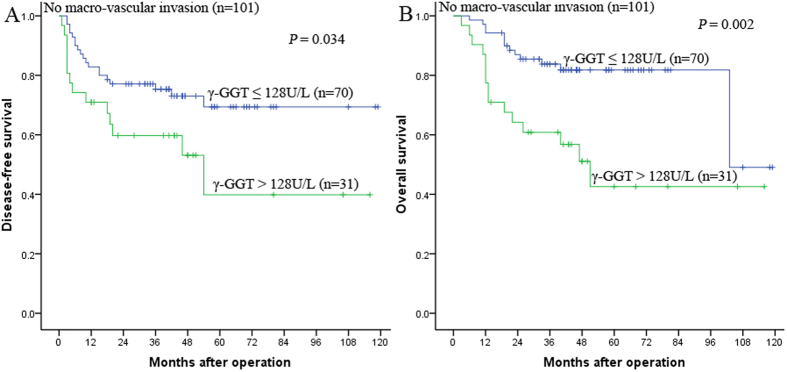
Kaplan-Meier survival curves demonstrating that patients with γ-GGT > 128 U/L exhibited shorter DFS (**A**) and OS (**B**) rates than those with γ-GGT ≤ 128 U/L in the no macro-vascular invasion subgroup (A: *P* = 0.034; B: *P* = 0.002, log-rank).

We further clarified the prognostic value of preoperative serum γ-GGT in HCC patients without macro-vascular invasion. Our results demonstrated that in the patients without macro-vascular invasion subgroup, the 1-, 3-, and 5-year DFS rates were 82.9%, 75.3%, and 69.4% for patients with γ-GGT ≤ 128 U/L and 71.0%, 59.8%, and 39.8% for patients with γ-GGT > 128 U/L, respectively (*P* = 0.034, Fig. 3A), and the 1-, 3-, and 5-year OS rates were 94.3%, 83.8%, and 81.8% for patients with γ-GGT ≤ 128 U/L and 77.4%, 60.8%, and 42.6% for patients with γ-GGT > 128 U/L, respectively (*P* = 0.002, Fig. 3B).

### Prognostic value of preoperative serum γ-GGT in HCC patients beyond the Milan criteria

**Figure 4:**
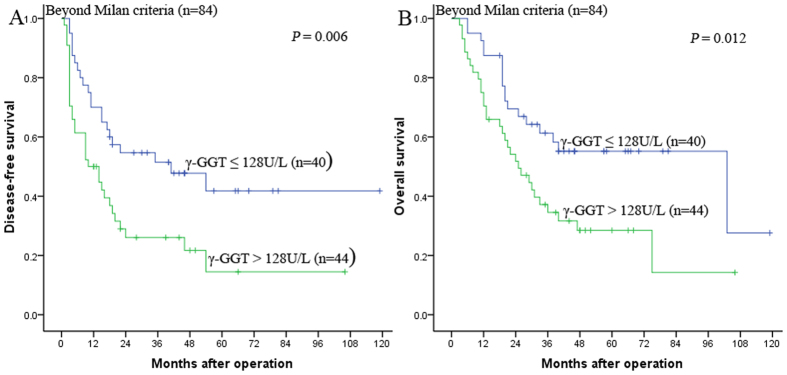
Kaplan-Meier survival curves demonstrating that patients with γ-GGT ≤ 128 U/L exhibited longer DFS (**A**) and OS (**B**) rates than those with γ-GGT > 128 U/L in the beyond the Milan criteria subgroup (A: *P* = 0.006; B: *P* = 0.012, log-rank).

The Milan criteria are recognized as the golden candidate selection criteria for HCC patients who receive liver transplantation. However, the Milan criteria are too restrictive and insufficient for the increasing list of candidates, and many patients beyond the Milan criteria receive LT. We further clarified the prognostic value of preoperative serum γ-GGT in HCC patients beyond the Milan criteria. Our results demonstrated that in the subgroup of patients beyond the Milan criteria, the 1-, 3-, and 5-year DFS rates were 70.0%, 51.4%, and 41.8% for patients with γ-GGT ≤ 128 U/L and 50.0%, 26.1%, and 14.5% for patients with γ-GGT > 128 U/L, respectively (*P* = 0.006, Fig. 4A), and the 1-, 3-, 5-year OS rates were 87.5%, 61.3%, and 55.2% for patients with γ-GGT ≤ 128 U/L and 70.5%, 34.5%, and 28.5% for patients with γ-GGT > 128 U/L, respectively (*P* = 0.012, Fig. 4B).

## Discussion

Liver transplantation is considered a curative treatment for HCC patients, especially those with underlying cirrhotic liver disease who are not eligible for hepatectomy. Unfortunately, HCC patients undergoing LT, which is designed to completely remove the tumor and the underlying liver disease, have a high recurrence rate due to the deposition of the recipient’s circulating tumor cells in the donor liver, lung, bone and subsequent HCC recurrence^28^. Hence, it is important to identify the risk factors for tumor recurrence. Several clinicopathological features including poor liver function, high serum AFP levels, large tumor size, multiple tumors, and macro and micro-vascular invasion have been previously reported to be indicators for poor prognosis in HCC patients^6^,^29^. The results of our study evidently demonstrate that elevated preoperative serum γ-GGT is a predictor of survival for HCC patients after LT.

γ-GGT is a crucial enzyme of glutathione (GSH) metabolism, and it is related to biotransformation, nucleic acid metabolism, and tumorigenesis^22^. γ-GGT has been widely used as a marker enzyme for several cancers, including ovarian tumors and renal cell carcinoma^18^,^19^. Recently, serum γ-GGT has been identified as a useful risk predictor in addition to traditional risk factors for cancer because it is a marker of oxidative stress^30^. Some studies initially focused on the possible association between serum γ-GGT and the incidence of various cancers^31^,^32^. More recently, elevated serum γ-GGT has been associated with a worse prognosis in many cancers, including renal cell carcinoma, esophageal squamous cell carcinoma and endometrial cancer^20^,^33^,^34^.

With respect to HCC, Zhang *et al.* firstly reported in 2011 that GGT levels were an important prognostic factor for patients with intermediate HCC treated with TACE in 2011, especially within the normal AFP subgroup^22^. Later, Guiu *et al.* also reported that GGT was an independent predictor of outcome after TACE for HCC in a European population^23^. Ma *et al.* reported that baseline serum GGT levels were a simple serum marker that may be used for prognosis in HCC treated by RFA^24^. We demonstrated that serum γ-GGT was a promising and reliable prognostic biomarker in HCC patients after hepatic resection, especially for patients with small HCC or AFP ≤ 200 ng/mL^21^. However, the prognostic value of serum γ-GGT in HCC patients treated by LT has not previously been reported. In the present study, we determined that the appropriate cut-off value of serum γ-GGT was 128 U/L for predicting recurrence. In the correlation analysis, we observed that preoperative serum γ-GGT > 128 U/L exhibited a strong connection with AFP > 400 ng/ml, larger tumor, and macro and micro-vascular invasion. The results predicted that elevated preoperative serum γ-GGT was significantly associated with advanced tumor stage and aggressive tumor behaviors in HCC. Our results indicated that the 1-, 3-, and 5-year DFS and OS rates in patients with γ-GGT ≤ 128 U/L group were higher than those with the γ-GGT > 128 U/L group, respectively. Preoperative serum γ-GGT was a risk factor for HCC patients who underwent liver transplantation. Furthermore, the multivariaret Cox proportional hazards model identified γ-GGT as an independent risk factor for HCC patients.

Macro-vascular invasion (portal or hepatic vein tumor thrombi) is a risk factor of poor prognosis in HCC patients^21^,^29^. However, some patients with no macro-vascular invasion suffer tumor recurrence shortly after LT. Therefore, it is important to identify simple and effective serum markers to predict the prognosis of HCC patients with no macro-vascular invasion. In the study, we determined that the prognosis of patients with γ-GGT > 128 U/L was worse than those with γ-GGT ≤ 128 U/L in the no macro-vascular invasion subgroup by stratification analysis. The result reveals that preoperative serum γ-GGT may predict the prognosis of HCC patients without macro-vascular invasion after LT, and suggests that HCC patients without macro-vascular invasion are more suitable for LT if they exhibited preoperative serum γ-GGT ≤ 128 U/L.

It is well known that the Milan criteria is the golden candidate selection criteria for HCC patients treated by LT^2^. However, it has been proven to be restrictive and far away from stratifying the growing candidate list. A large proportion of HCC patients beyond the Milan criteria have a potential curative chance and good outcome after LT. The primary cause of failure of the pretransplant diagnosis and staging by Milan criteria is the failure to account for tumor biology. In this study, stratification analysis of the Milan criteria revealed that the DFS and OS rates in the patients with γ-GGT ≤ 128 U/L were greater than those in the patients with γ-GGT > 128 U/L in the beyond the Milan criteria subgroup. The result suggests that preoperative serum γ-GGT levels may be used as a simple and effective marker for selecting HCC patients who are planning to undergo liver transplantation and are beyond the Milan criteria.

Several additional limitations of the study merit mention. Due to its retrospective nature and the relatively small size in a single center, we couldn’t divide the data into a training set and a test set for statistical validation. Further multicenter, large prospective studies are needed to confirm our findings. Although elevated preoperative serum γ-GGT predicted poor prognosis, we were not able to select liver transplantation candidates according to preoperative serum γ-GGT levels, which we hope to demonstrate in the future clinical trials.

In conclusion, our results demonstrate that elevated preoperative serum γ-GGT is significantly associated with advanced tumor stage and aggressive tumor behaviors, and that preoperative serum γ-GGT can be considered a prognostic factor for HCC patients after LT, especially for patients beyond the Milan criteria and with no macro-vascular invasion. Based on our findings, preoperative serum γ-GGT levels can be considered a simple and effective marker for the selection of HCC patients, who are candidates for liver transplantation, particular those beyond the Milan criteria.

## Additional Information

**How to cite this article**: Fu, S.-J. *et al.* Elevated Preoperative Serum Gamma-glutamyltranspeptidase Predicts Poor Prognosis for Hepatocellular Carcinoma after Liver Transplantation. *Sci. Rep.* **6**, 28835; doi: 10.1038/srep28835 (2016).

## Figures and Tables

**Table 1:** Relationship between preoperative serum γ-GGT levels and clinicopathological characteristics.

Category	Subcategory	Cases	γ-GGT (U/L)		*P* value
			≤128(n = 77)	>128(n = 53)	
Gender	Male	121	70	51	
	Female	9	7	2	0.241
Age (years)	≤50	68	42	26	
	<50	62	35	27	0.538
HBsAg	Positive	119	70	49	
	Negative	11	7	4	0.756
Child-Pugh stage	A	84	46	38	
	B	36	22	14	
	C	10	9	1	0.097
Preoperative tumor therapy	Yes	59	35	24	
	No	71	42	29	0.985
AFP (ng/ml)	≤400	82	57	25	
	>400	48	20	28	0.002
Size of largest tumor (cm)	≤5	74	55	19	
	5 to 8	22	10	12	
	>8	34	12	22	<0.001
Tumor number	≤3	93	58	35	
	>3	37	19	18	0.249
Edmondson grading	I–II	86	54	32	
	III–IV	44	23	21	0.248
Macro-vascular invasion	Yes	29	7	22	
	No	101	70	31	<0.001
Micro-vascular invasion	Yes	20	5	15	
	No	110	72	38	0.001
Milan criteria	Within	46	37	9	
	Beyond	84	40	44	<0.001
UCSF criteria	Within	69	49	20	
	Beyond	61	28	33	0.004
Hangzhou criteria	Within	81	59	22	
	Beyond	49	18	31	<0.001

γ-GGT, γ-Gamma-glutamyltransferase; HBsAg, Hepatitis B surface antigen; AFP, Alpha fetoprotein.

**Table 2:** Influence of clinicopathological characteristics on patient prognosis.

Variables	n	DFS				OS			
		1-yr	3-yr	5-yr	*P*	1-yr	3-yr	5-yr	*P*
Gender									
Male	121	71.1%	56.0%	47.2%		85.1%	63.4%	56.5%	
Female	9	77.8%	66.7%	66.7%	0.382	88.9%	77.8%	77.8%	0.539
Age (years)									
≤50	68	64.7%	52.1%	42.6%		80.9%	57.7%	49.0%	
>50	62	79.0%	61.8%	54.9%	0.152	90.3%	71.7%	67.2%	0.060
HBsAg									
Positive	119	72.3%	57.9%	49.1		85.7%	65.6%	58.5%	
Negative	11	63.6%	42.4%	42.4%	0.402	81.8%	63.6%	54.5%	0.780
Child-Pugh stage									
A	84	66.7%	48.7%	39.3%		83.3%	60.9%	56.0%	
B	36	80.6%	69.0%	63.3%		74.1%	67.7%	54.4%	
C	10	80.0%	80.0%	80.0%	0.037	80.0%	80.0%	80.0%	0.364
Preoperative tumor therapy									
Yes	59	72.9%	53.1%	50.7%		86.4%	66.3%	52.6%	
No	71	70.4%	59.8%	52.0%	0.580	84.5%	62.8%	60.9%	0.960
AFP (ng/ml)									
≤400	82	84.1%	72.5%	60.7%		92.7%	77.5%	70.8%	
>400	48	75.0%	29.6%	26.3%	<0.001	72.9%	41.7%	36.4%	<0.001
Size of largest tumor (cm)									
≤5	74	86.5%	75.3%	71.2%		95.9%	84.2%	78.6%	
5 to 8	22	68.2%	38.4%	25.6%		77.3%	54.2%	36.9%	
>8	34	41.2%	26.7%	16.7%	<0.001	67.6%	29.4%	29.4%	<0.001
Tumor number									
≤3	93	81.7%	69.1%	59.8%		87.1%	77.1%	68.3%	
>3	37	45.9%	25.3%	20.2%	<0.001	81.1%	31.3%	31.3%	<0.001
Edmondson grading									
I–II	86	73.3%	55.1%	46.4%		87.2%	65.9%	55.7%	
III–IV	44	68.2%	59.1%	51.7%	0.812	81.8%	61.1%	61.1%	0.799
Macro-vascular invasion									
Yes	29	44.8%	8.0%	4.0%		72.4%	23.6%	19.7%	
No	101	79.2%	70.7%	61.7%	<0.001	89.1%	76.7%	69.6%	<0.001
Micro-vascular invasion									
Yes	20	50.0%	20.8%	13.9%		75.0%	27.3%	21.8%	
No	110	75.5%	63.0%	54.7%	<0.001	87.3%	71.1%	64.7%	<0.001
γ-GGT (U/L)									
≤128	77	81.8%	70.7%	63.4%		93.5%	78.6%	75.1%	
>128	53	56.6%	35.5%	32.0%	<0.001	73.6%	43.8%	34.3%	<0.001
Milan criteria									
Within	46	93.5%	88.7%	85.3%		97.8%	95.6%	89.9%	
Beyond	84	59.5%	38.5%	28.5%	<0.001	78.6%	47.3%	40.8%	<0.001
UCSF criteria									
Within	69	91.3%	80.4%	78.1%		94.2%	89.5%	81.9%	
Beyond	61	49.2%	29.3%	15.9%	<0.001	75.4%	35.8%	31.3%	<0.001
Hangzhou criteria									
Within	81	87.7%	80.9%	71.8%		92.6%	84.8%	79.9%	
Beyond	49	44.9%	16.3%	10.2%	<0.001	73.5%	31.4%	24.2%	<0.001

DFS, disease-free survival; OS, overall survival; Other abbreviations as in Table 1.

**Table 3:** Prognostic factors for DFS and OS by multivariate Cox proportional hazards regression model.

Variables	DFS			OS		
	HR	95% CI	*P*	HR	95% CI	*P*
Child-Pugh stage	0.563	0.327–0.970	0.038			
Tumor number	2.632	1.541–4.494	<0.001	2.032	1.125–3.671	0.019
Size of largest tumor	1.461	1.058–2.016	0.021	1.906	1.373–2.647	<0.001
AFP	1.965	1.093–3.533	0.024			
γ-GGT	2.000	1.160–3.448	0.013	2.239	1.251–4.006	0.007

HR, hazard ratio; CI, confidence interval. Other abbreviations as in Tables 1 and 2.
